# Duration of antibiotic treatment and risk of recurrence after surgical management of orthopaedic device infections: a multicenter case-control study

**DOI:** 10.1186/s12891-019-2574-4

**Published:** 2019-05-01

**Authors:** Romy Spitzmüller, Denis Gümbel, Claas Güthoff, Sarah Zaatreh, Annett Klinder, Matthias Napp, Rainer Bader, Wolfram Mittelmeier, Axel Ekkernkamp, Axel Kramer, Dirk Stengel

**Affiliations:** 1 Department of Trauma, Reconstructive Surgery and Rehabilitation Medicine, University Medicine Greifswald, Ferdinand-Sauerbruch-Str, 17475 Greifswald, Germany; 2 Department of Trauma and Orthopaedic Surgery, BG Hospital Unfallkrankenhaus Berlin gGmbH, Warener Str 7, 12683 Berlin, Germany; 3 Center for Clinical Research, BG Hospital Unfallkrankenhaus Berlin gGmbH, Warener Str 7, 12683 Berlin, Germany; 4 Department of Orthopaedics, University Medicine Rostock, Doberaner Str 142, 18057 Rostock, Germany; 5 Department of Hygiene and Environmental Medicine, University Medicine Greifswald, Walther-Rathenau-Str 49A, 17489 Greifswald, Germany; 6 BG Kliniken Group of Hospitals, Leipziger Pl 1, 10117 Berlin, Germany

**Keywords:** Implant-associated infections, Antibiotic treatment, Reinfection, Orthopaedic devices

## Abstract

**Background:**

Device-related infections in orthopaedic and trauma surgery are a devastating complication with substantial impact on morbidity and mortality. Systemic suppressive antibiotic treatment is regarded an integral part of any surgical protocol intended to eradicate the infection. The optimal duration of antimicrobial treatment, however, remains unclear. In a multicenter case-control study, we aimed at analyzing the influence of the duration of antibiotic exposure on reinfection rates 1 year after curative surgery.

**Methods:**

This investigation was part of a federally funded multidisciplinary network project aiming at reducing the spread of multi-resistant bacteria in the German Baltic region of Pomerania. We herein used hospital chart data from patients treated for infections of total joint arthroplasties or internal fracture fixation devices at three academic referral institutions. Subjects with recurrence of an implant-related infection within 1 year after the last surgical procedure were defined as case group, and patients without recurrence of an implant-related infection as control group. We placed a distinct focus on infection of open reduction and internal fixation (ORIF) constructs. Uni- and multivariate logistic regression analyses were employed for data modelling.

**Results:**

Of 1279 potentially eligible patients, 269 were included in the overall analysis group, and 84 contributed to an extramedullary fracture-fixation-device sample. By multivariate analysis, male sex (odds ratio [OR] 2.06, 95% confidence interval [CI] 1.08 to 3.94, *p* = 0.029) and facture fixation device infections (OR 2.05, 95% CI 1.05 to 4.02, *p* = 0.036) remained independent predictors of reinfection. In the subgroup of infected ORIF constructs, univariate point estimates suggested a nearly 60% reduced odds of reinfection with systemic fluoroquinolones (OR 0.42, 95% CI 0.04 to 2.46) or rifampicin treatment (OR 0.41, 95% CI 0.08 to 2.12) for up to 31 days, although the width of confidence intervals prohibited robust statistical and clinical inferences.

**Conclusion:**

The optimal duration of systemic antibiotic treatment with surgical concepts of curing wound and device-related orthopaedic infections is still unclear. The risk of reinfection in case of infected extramedullary fracture-fxation devices may be reduced with up to 31 days of systemic fluoroquinolones and rifampicin, although scientific proof needs a randomized trial with about 1400 subjects per group. Concerted efforts are needed to determine which antibiotics must be applied for how long after radical surgical sanitation to guarantee sustainable treatment success.

## Background

Surgical site infections (SSI), including deep, implant-associated infections, remain serious complications in orthopaedic surgery, with marked impact on morbidity and mortality. They often result in multiple revisions, poor functional and health-related outcomes, and significant financial burden to health care systems worldwide [1–6].

Joint arthroplasty registries have greatly contributed to the knowledge about the risk of infection in high-income countries. Various definitions of SSI, follow-up intervals, and effect modifiers like age, co-morbidity, treatment indication, type of components etc. notwithstanding, national benchmarks typically range from 0.5 to 2.0% in primary, elective total hip and knee replacement [7–10]. Revision arthroplasty, however, is associated with a more than twofold risk of infection [9, 11–13].

In contrast, SSI after fracture fixation are far less understood, and registry or other large-scale data are generally missing. Numerous, often combined approaches are used for surgical fracture stabilization (e.g., primary external fixation followed by open reduction and internal fixation [ORIF], intramedullary nail fixation plus ORIF for a fracture of the distal fibula, ORIF of the tibia plafond plus nail of the fibula, etc.) conditional on the individual anatomical site, joint and soft tissue compromise, individual patient profiles, and so on. Plate and screw constructs are obviously at the highest risk of infection, even if ORIF is performed in a minimally-invasive fashion. The reported average SSI incidence rate after surgical fixation of lower extremity fractures is about 5% [14–22]. Yet, in case of open fractures and severe soft tissue injury, the risk of infection with both extra- and intramedullary fixation may far exceed 10% [23–25].

Key principles of preventing surgical site and implant-associated infections comprise proper skin disinfection [26], peri-operative antibiotic prophylaxis [27], use of antibiotic-loaded cements in hip and knee arthroplasty [28], intra-operative warming [29], irrigation of fracture wounds [25] and many others. In elective arthroplasty, current care bundles include pre-operative screening for MRSA carriage [30] and eradication of staphylococci by nasal mupirocin ointment and body washing [31–34]. In orthopaedic trauma, adequate timing of definitive fracture fixation (i.e., after damage-control, if necessary) [35, 36] remains a mainstay of preventing deep infections and other complications. Minimally-invasive access routes and biologically designed implants may further decrease the risk of infection because of less trauma to surrounding soft tissues, thereby avoiding a surgical second hit [37]. While all these interventions proved effective in reducing the incidence of SSI [38–40], the total number of infections is expected to rise globally because of an increasing number of orthopaedic procedures performed on an aging population with multiple risk factors (e.g., obesity, nicotine abuse, diabetes mellitus and other chronic diseases) [2, 41].

Deep SSI, specifically implant-related infections, demand aggressive surgical management. Decisive factors are the type of implant (i.e., arthroplasty or fracture fixation device), early or late onset of infection, stability etc., conditionally prompting serial debridement, one- or two-stage revision, septic arthrodesis, or complex reconstruction including muscle flaps or other types of tissue transfer [42–45]. Regardless of the particular device and primary indication for its use, systemic antibiotics are widely regarded a mainstay in treating implant-associated orthopaedic infections of any cause, although their role is currently not fully understood. As it is consensus that most available systemic antimicrobials cannot penetrate biofilms and destroy sessile bacteria, they may suppress or kill pathogens released from implant surfaces in their planktonic state [42–44, 46]. Current practice guidelines and expert consensus statements recommend systemic antibiotics to be applied for 2 weeks up to 3 months with or following a clear, potentially curative surgical concept [43, 44]. As antibiotic treatment potentially induces microbial resistance and causes adverse events, it must be as short as possible but as long as necessary.

The aim of the present multicenter case-control study was to investigate the influence of the duration of antibiotic treatment on the rate of recurrence (i.e., treatment failure) after surgical management of various orthopaedic device-related infections. Give the paucity of data and absence of clear-cut recommendations in this setting, we placed a focus on infected osteosynthesis constructs, namely extramedullary implant infections.

## Methods

### General remarks

This was a multi-institutional case-control study conducted at three academic tertiary referral hospitals in Germany (i.e., one university supra-regional trauma center, one maximum-care university orthopaedic joint replacement facility, and one supra-regional trauma center of the Federal Statutory Accident Insurance). This investigation was part of a larger network project aiming at preventing the spread of multi-resistant bacteria in the German Baltic region and Federal State of Mecklenburg-West-Pomerania, as funded by the German Ministry of Education and Research (BMBF). Details of the project can be assessed at http://www.hicare.de. All individual project modules, including this investigation, received ethical approval by the central and local Institutional Review Boards (IRB), and fully complied with national and European laws of data safety and protection.

### Research question

We posed the following primary research question in a PICOT (patient and problem, intervention, control, outcome, time) format:

*“In patients with an infected total joint arthroplasty or fracture fixation device demanding surgical revision, does the duration of systemic antimicrobial treatment impact the risk of re-infection within one year after the final surgical procedure?”*

We decided to break down the overall dataset to answer the following, more specific secondary PICOT question:

*“In patients with an infected extramedullary fracture fixation device demanding surgical revision, does the duration of systemic antimicrobial treatment impact the risk of re-infection within one year after the final surgical procedure?”*

### Definition of cases and controls

We defined cases as patients who sustained any reinfection demanding any surgical revision ≤1 year after the index procedure. Controls were defined as patients who did not sustain any infection demanding surgical revision (or any surgical revision for infection) ≤1 year, being aware the control status is fragile and may change to a case status with longer follow-up intervals. Given the catchment area and healthcare mandate of recruiting centers we presumed that patients normally followed-up as outpatients who did no longer show up or were admitted for reinfection within 1 year represented controls.

### Data acquisition and storage

Hospital databases were searched for eligible patients with the diagnosis of infection of total joint replacements or fracture fixation devices between 1999 and 2014 using ICD-10 codes T84.5 to 84.7 (complications caused by orthopaedic arthroplasties or implants) and M86.0 to M86.9 (osteomyelitis). Individual electronic and paper hospital charts were thoroughly reviewed to identify patients with an infection of a total hip or knee joint replacement, or infection of an osteosynthesis construct of the humerus, forearm, femur, tibia, fibula, ankle, or calcaneus were included in the study. Apart from ≥1 documented surgical procedure intended to cure the initial and reinfection (e.g., one- or two-stage revision with or without component retention or exchange, implant removal etc.) [47, 48], diagnostic criteria were documented clinical signs (e.g., warming, swelling, fever), presence of a sinus tract, inflammatory markers in peripheral blood (e.g., C-reactive protein [CRP], white blood cell count), and positive microbiological cultures of a surgically obtained specimen. Sonication of explanted hardware and advanced molecular methods (e.g., PCR) contributed to the diagnosis of infection, although we did not investigate this in detail. Patients treated non-operatively, those who underwent amputation or died within 1 year after the last revision were excluded from the study. Patient demographics, risk profiles, details on infection and reinfection, surgical procedures, and antibiotic treatment were stored in a professional electronic data capture system (secuTrial®, interActive Systems GmbH, Berlin, Germany).

### Statistical analysis

The primary endpoint of this study was reinfection within 1 year after the last surgical procedure intended to cure or control the primary infection. The primary exposition variable was duration of antibiotic treatment (number of days between the last surgical procedure and the last day of antibiotic medication). Due to its variance, it was dichotomized into “administration of systemic antibiotics <14 days” and “administration of systemic antibiotics ≥14 days”. Secondary exposition variables were patient demographics, comorbidity (e.g., smoking, diabetes mellitus), American Society of Anesthesiologists physical status (ASA) classification, type of implant, microbial spectrum, local antibiotic treatment, antibiotics used, doses and routes of administration.

We provided raw numbers and percentages for categorical data. Continuous measures were expressed as means with standard deviations (SD) or medians with interquartile ranges (IQR), based on the underlying distribution and skewness. Where appropriate, we employed the chi^2^ or Kruskal-Wallis test for bivariate, exploratory group comparisons.

Uni- and multivariate logistic regression analysis were used for modelling the association between primary and secondary exposition variables and endpoints. Exposure variables with univariate *p*-values < 0.2 and variables with a confounding effect were included in the model. To test possible confounding effects of secondary exposition variables, each of these variables were assessed by single logistic regression analyses. If unadjusted odds ratios (OR) differed from adjusted OR by 10%, the secondary exposition variable was included in the model. We also used exact logistic regression analysis as a computationally more intensive tool which is regarded to provide more robust estimates in case of small sample sizes and skewed distributions [49].

For multivariate analysis in the infected fracture-fixation device scenario, age was categorized into quartiles. Days of systemic exposure to fluoroquinolones and rifampicin were clustered in clinically meaningful categories with comparable sample sizes. A first-order interaction term was introduced respecting combined fluoroquinolone-rifampicin antimicrobial therapy. Model fit and explained variance was assessed by the area under the receiver operating characteristics curve (AUC / ROC).

OR were calculated and reported with 95% confidence intervals (CI). Results with *p*-values < 0.05 may be interpreted as incompatible with chance, although we did not go for inferential testing here. Statistical analysis employed SPSS (V22.0, IBM SPSS Statistics) and STATA (V14.0, Stata Corp., TX, USA).

## Results

In total, electronic hospital databases revealed 1279 patients potentially eligible for this study. The screening and selection process resulted in complete data sets from 269 patients (59 cases, 210 controls) to be included in the statistical analysis. Of those, 84 (31 cases, 53 controls) had infections of extramedullary fracture fixation devices.

**Fig. 1:**
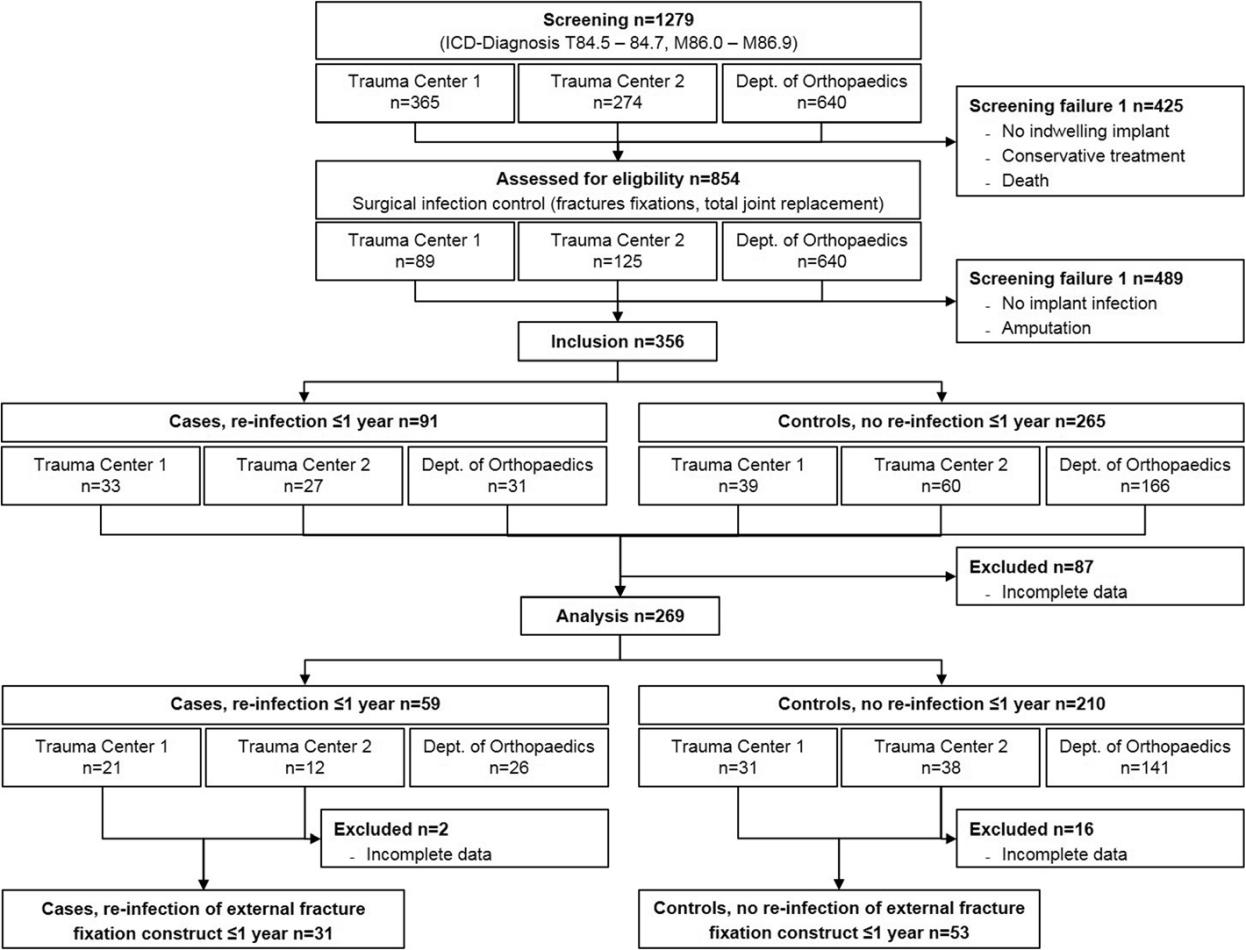
STROBE flowchart of the patient selection process and assignment to case and control groups

Thus, we roughly had a an overall ratio of cases to controls of 1: 4, and a 1: 2 pairing with regard to infected ORIF constructs. The study profile according to STROBE recommendations is shown in Fig. 1.

Sociodemographic and clinical characteristics of the eligible sample at the time of first revision are presented in Table 1. Case patients were more likely to be male smokers with an infected fracture fixation device. Most frequently prescribed antibiotics were cefuroxime, moxifloxacin, and clindamycin. Systemic antibiotic treatment up to 14 days was applied to 22 (37%) case and 109 (52%) control subjects. Correspondingly, 37 cases (63%) and 101 controls (48%) received prolonged treatment for ≥14 days.

**Table 1:** Baseline profile, entire study population

Variable	Cases	Controls
n	59	210
Median age, years (IQR)	63 (48–71)	67 (55–73)
Gender, n (%)		
Male	42 (71)	106 (50)
Female	17 (29)	104 (50)
Median BMI (IQR)	28 (25–32)	28 (25–33)
Smoker, n (%)	20 (34)	42 (20)
Diabetes mellitus, n (%)	10 (17)	49 (23)
ASA status, n (%)		
1	7 (12)	18 (9)
2	28 (47)	98 (47)
≥ 3	24 (41)	94 (45)
Type of implant		
Total joint arthroplasty	28 (47)	157 (75)
Fracture fixation device	31 (53)	53 (25)
Infection site		
Lower extremity	56 (95)	200 (95)
Upper extremity	3 (5)	10 (5)
MRSA present, n (%)	3 (5)	6 (3)
Preferred antibiotics, n (%)		
Cefuroxime	19 (32)	106 (50)
Moxifloxacin	13 (22)	66 (31)
Clindamycin	7 (12)	38 (18)

*IQR* interquartile range, *BMI* Body Mass Index, *ASA* American Society of Anesthesiologists physical status classification system, *MRSA* methicillin-resistant *staphylococcus aureus* spec

Univariate analysis suggested an increased risk of recurrent infection with longer antibiotic treatment (OR 1.82, 95% CI 1.00 to 3.28, *p* = 0.049). Male gender (OR 2.42, 95% CI 1.30 to 4.53, *p* = 0.005), smoking (OR 2.05, 95% CI 1.09 to 3.88, *p* = 0.027), and infection of a fracture fixation device (OR 2.25, 95% CI 1.21 to 4.2, *p* = 0.032) were associated with an increased likelihood of developing relapse. In the multivariate model, male sex (OR 2.06, 95% CI 1.08 to 3.94, *p* = 0.029) and fracture fixation device infections (OR 2.05, 95% CI 1.05 to 4.02, *p* = 0.036) remained predictors of reinfection. The odds of recurrence of implant-related infections was 1.85 higher for patients with antibiotic treatment lasting ≥14 days than for those with treatment shorter than 14 days (OR 1.85, 95% CI 0.99 to 3.48, *p* = 0.055).

Table 2 shows the profile of the patient subsample with extramedullary fracture device-associated infections. Altogether, key baseline criteria were well balanced across cases and controls. None of them were associated with the risk of recurrent infection by univariate analysis (Table 3). Univariate point estimates suggested a nearly 60% reduced odds of re-infection with systemic fluoroquinolones (OR 0.42, 95% CI 0.04 to 2.46) or rifampicin treatment (OR 0.41, 95% CI 0.08 to 2.12) for up to 31 days, although the width of confidence intervals prohibited robust statistical or clinical inferences.

**Table 2:** Baseline profile, infected ORIF constructs only

Variable	Cases	Controls	*p*
n	31	53	
Median age, years (IQR)	55 (45–61)	51 (37–67)	0.849
Gender, n (%)			0.706
Male	20 (65)	32 (60)	
Female	11 (35)	21 (40)	
Median BMI (IQR)	26.4 (24.2–32.4)	26.9 (23.8–30.3)	0.513
Smoker, n (%)	12 (39)	18 (34)	0.661
Diabetes mellitus, n (%)	3 (10)	4 (8)	0.733
ASA status, n (%)			
1	6 (19)	11 (21)	0.890
2	16 (52)	28 (53)	
≥ 3	7 (23)	12 (23)	
unknown	2 (6)	2 (4)	
Infection site			0.487
Lower leg	26 (84)	41 (77)	
Femur	2 (6)	8 (15)	
Upper extremity	3 (10)	4 (8)	
Additional IM nail, n (%)	2 (6)	7 (13)	0.334
Microbiology, n (%)			
MSSA	21 (68)	24 (45)	0.046
MRSA	3 (10)	3 (6)	0.490
CNS	2 (6)	11 (21)	0.080
Enterococci	3 (10)	12 (23)	0.134
Streptococci	3 (10)	3 (6)	0.490
E. coli	1	3	
Enterobacter spec.	3	2	
Corynebacter spec.	1	1	
Proteus spec.	0	1	
Pseudomonas spec.	2	2	
Peptostreptococci	0	3	
Klebsiella spec.	0	2	
Candida spec.	0	1	
Local gentamicin, n (%)	6 (19)	10 (19)	0.956
Systemic antibiotics, n (%)			
Fluoroquinolones	13 (42)	23 (43)	0.896
Rifampicin	8 (26)	15 (28)	0.805
Cefuroxime	6 (19)	14 (26)	0.463
Aminopenicillins	9 (29)	9 (17)	0.194
Clindamycin	2 (6)	5 (9)	0.633
Linezolid	1	1	
Vancomycin	0	2	
Gentamicin	0	2	
Imipenem	1	1	
Tazobactam	1	2	
Combined fluoroquinolones and rifampicin			
None	29 (55)	18 (58)	
Any	10 (19)	5 (16)	
Fluoroquinolones and / or rifampicin	14 (26)	8 (26)	

*IQR* interquartile range, *BMI* Body Mass Index, *ASA* American Society of Anesthesiologists physical status classification system, *MSSA* methicillin-sensitive *staphylococcus aureus* spec, *MRSA* methicillin-resistant *staphylococcus aureus* spec, *CNS* coagulase-negative staphylococci

**Table 3:** Results of univariate logistic regression and exact logistic regression analysis Baseline profile, infected ORIF constructs only

Variable	Logistic regression, OR (95% CI)	*p*	Exact logistic regression, OR (95% CI)	*p*
Age	1.01 (0.98–1.03)	0.641	1.01 (0.98–1.03)	0.650
Male gender	1.19 (0.48–2.99)	0.706	1.19 (0.44–3.35)	0.889
BMI	1.02 (0.96–1.08)	0.598	1.02 (0.95–1.08)	0.601
Smoking	1.23 (0.49–3.08)	0.661	1.23 (0.44–3.38)	0.836
Diabetes mellitus	1.31 (0.27–6.29)	0.734	1.31 (0.18–8.35)	1.000
ASA class	1.03 (0.52–2.04)	0.924	1.03 (0.49–2.17)	1.000
MRSA present	1.79 (0.34–9.45)	0.495	1.77 (0.22–14.15)	0.779
Local antibiotics	1.03 (0.33–3.18)	0.956	1.03 (0.27–3.59)	1.000
Systemic fluoroquinolones				
Any use	0.94 (0.38–2.31)	0.896	0.94 (0.35–2.53)	1.000
1 to 7 days	1.04 (0.30–3.68)	0.949	1.04 (0.23–4.29)	1.000
8 to 31 days	0.42 (0.08–2.18)	0.300	0.42 (0.04–2.46)	0.499
> 31 days	1.43 (0.41–4.92)	0.572	1.42 (0.34–5.85)	0.796
1 to 31 days	0.73 (0.25–2.11)	0.560	0.73 (0.21–2.35)	0.758
> 31 days	1.43 (0.41–4.92)	0.572	1.42 (0.34–5.85)	0.796
Systemic rifampicin				
Any use	0.88 (0.32–2.40)	0.805	0.88 (0.28–2.64)	1.000
1 to 31 days	0.41 (0.08–2.12)	0.289	0.42 (0.04–2.35)	0.476
> 31 days	1.42 (0.42–4.74)	0.572	1.41 (0.35–5.60)	0.790
Fluoroquinolones or rifampicin	0.95 (0.56–1.59)	0.838		
Either	0.81 (0.24–2.74)	0.729	0.81 (0.19–3.13)	0.980
Combined	0.92 (0.32–2.63)	0.877	0.92 (0.28–2.94)	1.000

*IQR* interquartile range, *BMI* Body Mass Index, *ASA* American Society of Anesthesiologists physical status classification system, *MRSA* methicillin-resistant *staphylococcus aureus* spec, *MSSA* methicillin-sensitive *staphylococcus aureus* spec, *MRSA* methicillin-resistant *staphylococcus aureus* spec, *CNS* coagulase-negative staphylococci

**Fig. 2:**
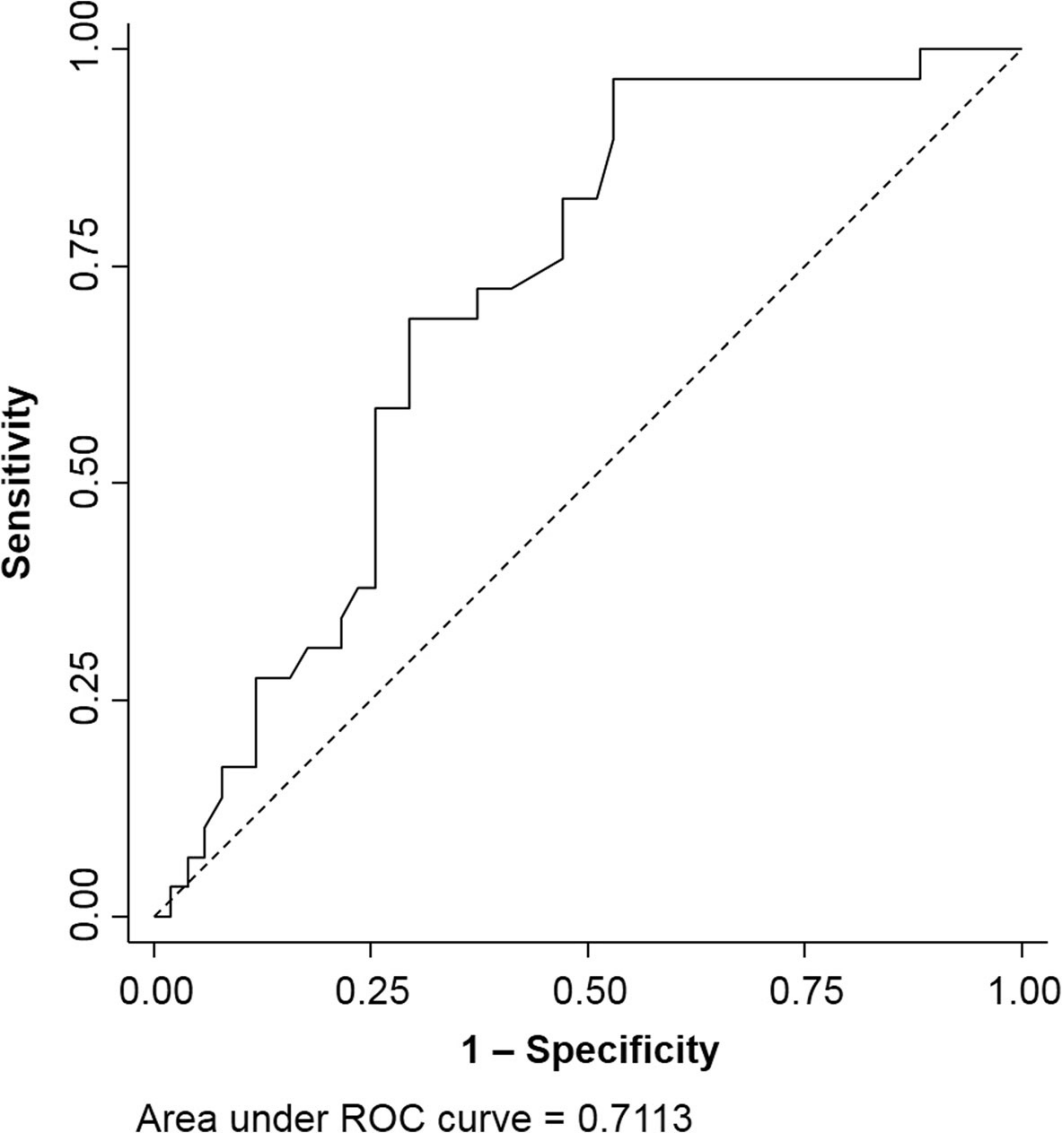
Explained variance of the risk of recurrent extramedullary fracture-fixation-device-related infections by the final logistic regression model (Table 4), expressed by the area under the receiver operating characteristics curve (AUC / ROC)

**Fig. 3:**
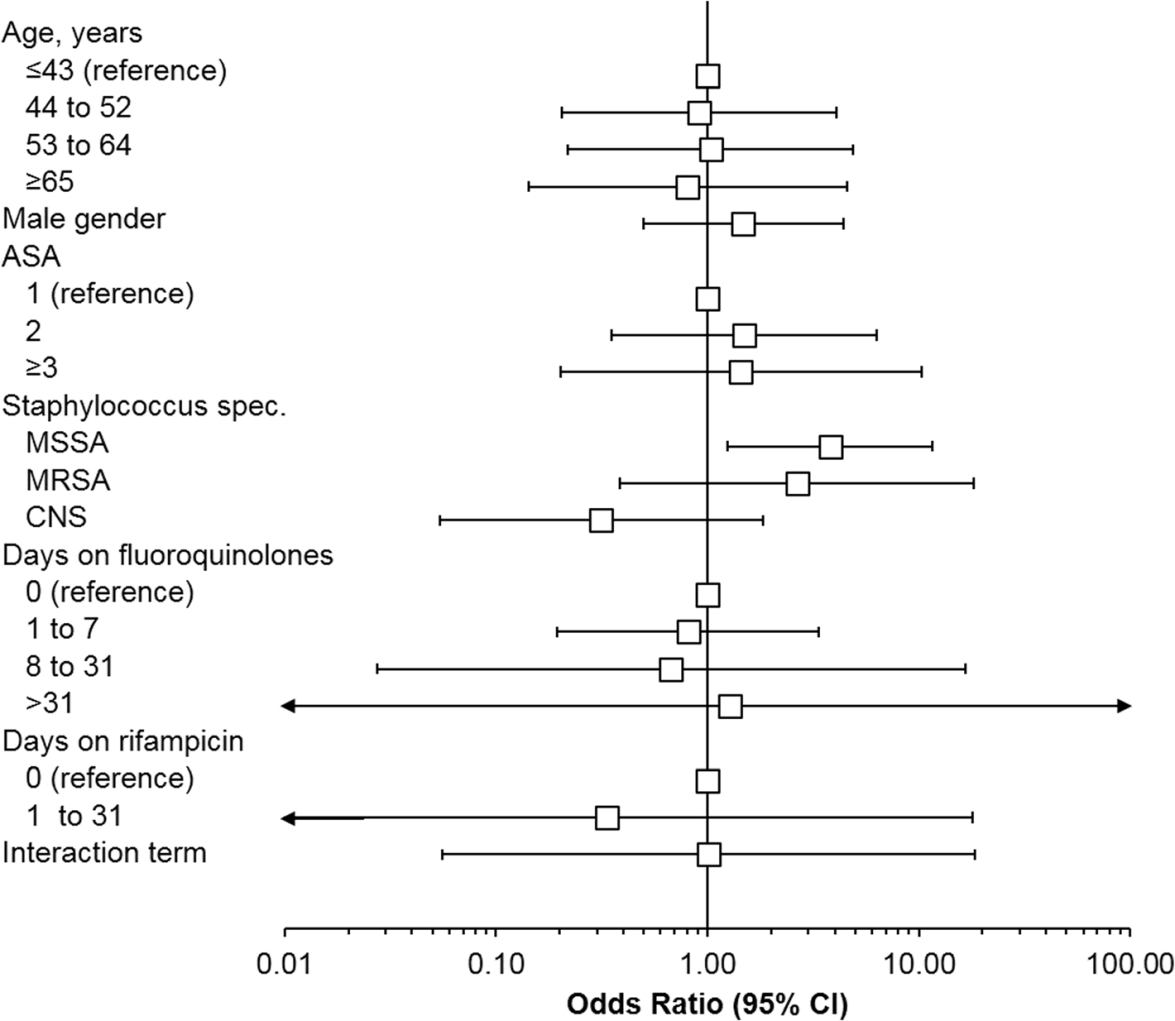
Forest plot of individual odds ratios (OR) and 95% confidence intervals (CI) obtained from the final multivariate logistic model (Table 4)

The best predictive multivariate model explained about 71% of infection recurrences and included age, gender, ASA class, the presence of various staphylococci, and exposure to systemic fluoroquinolones and rifampicin (Table 4 and Fig. 2). Individual OR are shown in a forest-plot format in Fig. 3.

**Table 4:** Results of multiivariate logistic regression regression analysis, infected ORIF constructs only

Variable	Logistic regression, OR (95% CI)	*p*
Age (quartiles)		
Male gender	0.94 (0.541–1.63)	0.641
ASA	1.52 (0.53–4.34)	0.706
MSSA present	1.26 (0.48–3.31)	0.598
MRSA present	3.84 (1.29–11.41)	0.661
CNS present	2.16 (0.36–13.13)	0.734
Duration of fluoroquinolone treatment (categorized)	0.31 (0.06–1.67)	0.924
Duration of rifampicin treatment (categorized)	0.67 (0.23–11.98)	0.495
Interaction	0.17 (0.01–5.45)	0.956

*ASA* American Society of Anesthesiologists physical status classification system, *MSSA* methicillin-sensitive *staphylococcus aureus* spec, *MRSA* methicillin-resistant *staphylococcus aureus* spec, *CNS* coagulase-negative staphylococci

## Discussion

In this case-control study, we explored a mixed population with orthopaedic device-associated infections for potential predictive variables of infection recurrence after attempted curative surgery. We had a particular interest in studying the influence of the duration of systemic antimicrobial therapy on infection relapse. In the overall sample, male gender, smoking, and infection of a fracture fixation device were associated with an increased risk of subsequent reinfection.

Smoking is a well-known, modifiable risk factor for SSI and recurrent infection in orthopaedic surgery, both in elective total joint arthroplasty [50–57] and fracture care [20, 24, 58, 59], and ORs observed here were in line with previous investigations. As men are more likely to be active smokers than women, this may, in part, contribute to the gender component observed in this and previous studies, although other (specifically genetic) effect modifiers need to be scrutinized in the near future [24, 60]. The risk of reinfection with an indwelling fracture fixation device was almost 2.3 times higher compared to total joint arthroplasty. This is in accordance with previous studies [13, 61, 62].

The best explanation for the observed increased risk of recurrence with longer systemic antibiotic therapy (i.e. > 14 days) is that doctors opted for prolonged suppressive antimicrobial treatment because of patient- and disease-related characteristics not accessible by hospital and administrative chart documentation. It must not be concluded from the present data that prolonged systemic antibiotic therapy increases the risk of recurrent infection. This leads to the major limitations of this study- it was retrospective, used hospital chart data, and, after applying predefined selection criteria, enrolled a rather small sample of patients.

We did not account for single- and two-stage revision protocols in infected total joint arthroplasties, and patients with subsequent reinfections may have consulted another hospital, thereby introducing verification bias.

We had deliberately chosen a case-control design as orthopaedic device-associated infections in the developed countries are serious but comparably rare events, and the incidence of reinfection after an index infection currently remains unpredictable. In this situation, a case-control strategy is generally more efficient than a cohort approach to estimate possible effect sizes but only allows for limited causal inference between exposure and outcome variables. A major lesion learned from this study is that it is almost impossible to derive scientifically precise and unequivocal information about process and outcome parameters in septic orthopaedic surgery from current hospital documentation systems. Detailed assessment of patients’ charts is necessary to obtain clinically relevant data, and to abstract the individual course from initial presentation over surgical procedures to discharge and follow-up.

Regarding the subsample of patients with infected fracture-fxation devices, both the presence of staphylococci as well as systemic treatment with fluoroquinolones and rifampicin for about 30 days predicted the risk of reinfection, although the sample size and study design did not allow for robust conclusions. Because of the small sample size, the final model also may have been saturated.

Answering the important question whether prolonged suppressive systemic antimicrobial treatment reduces the risk of reinfection clearly demands a multicenter RCT. This, however, needs to be conducted under EMA (European Medicines Agency), FDA (US Food and Drug Administration), and other international pharmaceutical regulations, with major requirements on trial logistics. Assuming a baseline risk of infection of 5% for any anatomical site, fracture severity, soft tissue compromise and so on, two-sided type I and II errors of 2% (accounting for multiple testing) and 15% (including a power reserve), respectively, a trial aiming at demonstrating a relative risk reduction of 50% by prolonged (i.e., ≥31 days) over shorter systemic antimicrobial treatment must recruit about 2 × 1400 patients, depending on the preferred adaptive design, number of interim analyses, strata etc. One may also think of a non-inferiority trial demonstrating that shorter systemic antibiotic treatment (e.g., 30 days) is not inferior to longer application with regard to infection relapse. This will, however, demand even higher sample sizes. We call for a European initiative to investigate the optimal duration of systemic antibiotic therapy for patients with fracture-fixation-device-associated infections undergoing revision surgery. This may, apart from standardizing diagnostic, peri-operative, surgical, and aftercare protocols, identify the most effective empiric systemic antibiotic regimen for patients suffering from deep SSI, namely device-related infections, adhering to the principles of antibiotic stewardship.

## Conclusion

In summary, up to 31 days of systemic exposure to fluoroquinolones and / or rifampicin appear to lower the risk of reinfection after surgical revision for infected extramedullary fracture-fixation constructs, although present results are compatible with chance. A large-scale confirmatory trial is needed to confirm that prolonged systemic suppressive antibiotic therapy is more effective than shorter administration in reducing reinfections, or that shorter treatment is not inferior to prolonged exposure in achieving this endpoint.
